# Amylin alters human brain pericyte viability and NG2 expression

**DOI:** 10.1177/0271678X16657093

**Published:** 2016-01-01

**Authors:** Nina Schultz, Elin Byman, Malin Fex, Malin Wennström

**Affiliations:** 1 Clinical Memory Research Unit, Department of Clinical Sciences, Lund University, Malmö, Sweden; 2 Unit for Molecular Metabolism, Lund University Diabetes Centre, Department of Clinical Sciences, Lund University, Malmö, Sweden

**Keywords:** Alzheimer’s disease, cell death mechanisms, diabetes, hippocampus, pericytes

## Abstract

Amylin, a pancreatic β-cell-derived peptide hormone, forms inclusions in brain microvessels of patients with dementia who have been diagnosed with type 2 diabetes and Alzheimer’s disease. The cellular localization of these inclusions and the consequences thereof are not yet known. Using immunohistochemical staining of hippocampus and parahippocampal cortex from patients with Alzheimer’s disease and non-demented controls, we show that amylin cell inclusions are found in pericytes. The number of amylin cell inclusions did not differ between patients with Alzheimer’s disease and controls, but amylin-containing pericytes displayed nuclear changes associated with cell death and reduced expression of the pericyte marker neuron-glial antigen 2. The impact of amylin on pericyte viability was further demonstrated in in vitro studies, which showed that pericyte death increased in presence of fibril- and oligomer amylin. Furthermore, oligomer amylin increased caspase 3/7 activity, reduced lysate neuron-glial antigen 2 levels and impaired autophagy. Our findings contribute to increased understanding of how aggregated amylin affects brain vasculature and highlight amylin as a potential factor involved in microvascular pathology in dementia progression.

## Introduction

Amylin, or islet amyloid polypeptide, is a pancreatic β-cell-derived peptide hormone, co-produced and co-secreted with insulin. The peptide is recognized as a regulator of glycogen release and appetite, but is also associated with pathogenic features of type 2 diabetes (T2D). Amylin accumulates, aggregates and forms depositions during pre-diabetic and early stages of T2D, when insulin and amylin are released at high levels. Amylin depositions are found in various organs, including the pancreas, heart tissue and kidneys of patients with T2D,^1^ where it is known to induce cell death.^2,3^ The specific signalling pathway underlying amylin-induced cell death is not yet fully understood, but a few mechanisms including impaired autophagy,^4^ oxidative stress^5,6^ and different types of membrane damage^7^ have been suggested.

Amylin readily crosses the blood–brain barrier (BBB)^8^ and binds to receptors in most brain areas including the memory-forming hippocampus.^9,10^ The crossing of amylin over the BBB has been demonstrated in a study in which peripherally injected radiolabelled amylin was detected in trace amounts in the rodent brain.^8^ The radiolabelled amylin was associated with capillaries to some extent and was found throughout the cortex, indicating that amylin, at least in part, crosses at the vasculature. A recent study has shown amylin depositions in the brain of patients with dementia with T2D and Alzheimer’s disease (AD).^1^ These findings indicate that amylin aggregation and accumulation similar to that seen in the periphery also occurs in the brain. The depositions were found mostly in brain vessel walls and in the brain parenchyma, where they co-localized with amyloid beta (Aβ) 40 and 42 depositions. Since these amyloid peptides are well-established hallmarks of cerebral amyloid angiopathy (CAA) and AD pathology, it was hypothesised that amylin could potentially be involved in AD pathology,^1^ a hypothesis justified by the increasing number of studies showing a similarity and interaction between Aβ and amylin.^1,9–11^ The impact of amylin on brain function has moreover been demonstrated in a preclinical study in which rats overexpressing human amylin showed neurological deficits and neuroinflammation.^12^

The amylin brain tissue study mentioned above showed that amylin, besides forming depositions in the vessel walls and brain parenchyma, also forms dense amylin inclusions/plaques in microvessels,^1^ but the cellular localization of these inclusions is still unknown. Given the potential impact of amylin on brain function and dementia progression, we find it important to reveal the identity of the microvessel-associated cells containing amylin inclusions and to further investigate the consequences of this. We therefore used immunohistological staining techniques to analyse microvessel-associated amylin cell inclusions in hippocampus and parahippocampal cortex (PHC) of patients with AD and non-demented controls (NCs). We discovered that amylin forms cell inclusions in pericytes and that these amylin-containing pericytes showed nuclear changes and loss of neuron-glial antigen 2 (NG2), a proteoglycan important for pericyte proliferation, migration and survival. We also used in vitro studies to demonstrate that amylin, dependent on aggregation form, has a direct impact on pericyte viability, morphology, autophagy, NG2 expression and caspase 3/7 activity.

## Materials and methods

### Brain samples

Samples of midlevel hippocampus and PHC from clinically and post-mortem verified patients with AD (n = 5) and non-demented controls (NC; n = 6) from the Netherlands Brain Bank (NBB) were pretreated and sectioned according to previously described procedures for successful NG2 immunostaining.^13^ Demographic data, APOE genotype and neuropathological assessment (Braak stages) of the individuals are presented in Table 1. None of the individuals showed alterations characteristic of CAA. Additional staining against Aβ_1–16_ (see Immunostaining procedures) confirmed the lack of CAA in the brain samples as well as the increase in Aβ plaques in the PHC and hippocampus in AD patients compared with NCs (Table 1). One NC and one patient with AD were diagnosed with T2D. Written informed consent for the use of brain tissue and clinical data for research purposes was obtained from all patients or their next of kin in accordance with the International Declaration of Helsinki. Medisch Ethische Toetsingscommissie (METc) of VU University has approved the procedures of brain tissue collection and the regional ethical review board in Lund has approved the study. All human data were analysed anonymously.

**Table 1.** Demographic data, apolipoprotein E genotype, neuropathological assessment and number of amylin inclusions in the hippocampus and the PHC of individuals included in the study.

Clinical diagnosis^a^	T2D	Gender	Age (years)	*APOE* ɛ genotype	Braak stages (Aβ/NFT/LB)^b^	Aβ-plaques ML^c^	Aβ-plaques PHC^c^	Amylin inclusions ML^d^	Amylin inclusions PHC^d^
AD		F	71	4/3	5/C/0	(++++)	(++++)	1	2
AD		F	64	4/4	4/C/0	(+++)	(+++)	1	1
AD		F	87	3/3	4/C/0	(++++)	(++++)	2	3
AD	+	M	74	4/3	4/C/6	(++++)	(++++)	15	12
AD		M	83	4/3	3/B/0	(++)	(+++)	4	1
NC		F	87	3/3	1/B/0	(–)	(+++)	6	2
NC	+	F	84	3/3	2/B/0	(++)	(++++)	14	19
NC		F	91	3/2	1/B/0	(+)	(+)	1	1
NC		F	83	3/2	1/B/0	(+)	(++)	3	0
NC		F	75	3/3	1/O/0	(–)	(–)	6	6
NC		M	55	3/3	0/O/0	(–)	(–)	2	2

a Individuals clinically diagnosed with Alzheimer’s disease (AD) and non-demented controls (NC) included in the study.

b Braak staging of amyloid beta (Aβ), neurofibrillary tangles (NFT) and Lewy bodies (LB).

c Plaque load is indicated either as (–) no Aβ plaques visible, or (+) low, (++) moderate and (++++) very high number of Aβ plaques.

d Mean number of amylin inclusions in representative fields of the hippocampal molecular layer (ML) and the III–IV cortical layer of the PHC.

### Immunostaining procedures

The sections were immunohistochemically (IHC) stained against amylin (rabbit-anti-amylin T4149 and T4157; Peninsula Laboratories, San Carlos, CA) or Aβ (mouse anti-Aβ_1-16_ 6E10; Covance, Princeton, NJ). The sections were quenched in 3% H_2_O_2_ and 10% methanol for 30 min and incubated in Impress reagent kit blocking solution (Vector Laboratories, Burlingame, RI) for 1 h at room temperature (RT), followed by incubation with primary antibodies in blocking solution overnight at 4℃. Sections were then incubated with the appropriate Ig Impress reagent kit secondary antibody (Vector Laboratories, Burlingame, RI) for 2 h at RT followed by peroxidase detection for 2 min (0.25 mg/ml diaminobenzidine and 0.012% H_2_O_2_). Amylin cell inclusions in microvessels in the molecular layer (ML) of hippocampus and the III–IV cortical layer of PHC were scored by two independent blinded observers using an Olympus AX70 light microscope equipped with 20× objectives. Pictures (Olympus cellSens Dimension) of five randomly chosen fields (345 × 440 µm) within ML and IV layer of PHC of three sections (15 fields in total) from each individual were scored. Values were averaged and presented as mean number cell inclusions per ML and PHC (Table 1). Aβ plaques in hippocampus and PHC were scored semi-quantitatively as none visible (−), low number (+), moderate number (++), high number (+++) and very high number (++++) of Aβ plaques (Table 1).

Cells containing amylin cell inclusions were identified by double immunofluorescence staining. Antibodies directed against pericyte marker NG2 (mouse anti-NG2; Millipore, Darmstadt, Germany), laminin (mouse anti-laminin; Dako, Glostrup, Denmark) or perivascular macrophages marker CD163 (mouse anti-CD163; AbD Serotec Raleigh, NC) were used in combination with rabbit anti-amylin T4149 antibody. Sections were incubated in 5% normal goat serum (Millipore, Darmstadt, Germany) for 1 h at RT, followed by incubation with primary antibodies in blocking solution overnight at 4℃. Sections were then incubated with Alexa 488-conjugated goat anti-mouse (Invitrogen, Carlsbad, CA) and biotin-conjugated goat anti-rabbit (Dako, Glostrup, Denmark) for 2 h at RT followed by incubation with Dylight 549-conjugated streptavidin (Vector Laboratories, Burlingame, RI) for 2 h at RT. Sections were then mounted with Vectashield Set mounting medium containing DAPI (Vector Laboratories, Burlingame, RI). The sections were analysed using an Olympus AX70 light microscope equipped with 4 40× objectives. Co-localization between amylin, DAPI, laminin or NG2 and cell nucleus morphology was analysed using confocal microscopy (Zeiss LSM 510; Zeiss, Oberkochen, Germany) and Zen 2009 software. Ten amylin cell inclusions (NC (n = 5) and AD patients (n = 5)) from each double staining (i.e. amylin/NG2 staining and amylin/laminin staining) were selected in the red channel (549 nm) and thereafter analysed for co-localization with NG2 or laminin (green channel). Amylin-containing pericytes were defined as cells with amylin cell inclusions enclosed by NG2 or laminin (Supplementary Figure 1(a) to (h)).

The NG2 immunoreactive intensity of amylin-containing pericytes (n = 20) and pericytes without amylin (n = 20) was determined by analysis (Zen 2009 software) of pictures taken in fluorescent light microscope (40× magnification). The amylin-containing pericytes were selected in the red channel (amylin) and thereafter pictures were taken in the green channel (NG2). Each picture comprised one amylin-containing pericyte and one pericyte without amylin. The NG2 intensity of each amylin-containing pericyte was compared with the intensity of non-amylin-containing pericyte within the same picture (see statistical analysis) to avoid potential fading differences between pictures.

### Cells

Primary fetal human brain vascular pericytes (HBVP) cells were bought from ScienCell Research Laboratories (Carlsbad, CA); the cells were obtained, in compliance with local, state and federal laws and regulations, from donors who signed informant consent. The HBVPs were grown in pericyte cell culture medium (PM) (ScienCell Research Laboratories, Carlsbad, CA) containing 2% fetal bovine serum. Cells were grown as monolayers in poly-l-lysine-coated 96-well plates (Sigma-Aldrich, St. Louis, MO) or eight-well chamber slides (Lab-Tek) in humified air with 5% CO_2_ at 37℃ until 50% confluent.

### Amylin preparations

Fibril- and oligomer-enriched preparations (EP) of amylin were prepared according to a previously published protocol.^14^ Amylin peptide (Bachem, Bubendorf, Switzerland) was dissolved in cold hexafluoro-2-propanol (Sigma-Aldrich, St. Louis, MO), aliquoted, speed-vacuum dried and stored at −80℃ until use. For preparations, amylin was solubilized in 10 mM NaOH (pH 11). The pH was adjusted to pH 7 by diluting the solution in phosphate buffer to a concentration of 100 μM. Fibril-EP was generated by incubating for 72 h at 37℃ with agitation, and oligomer-EP were generated by incubating for 20 min with agitation at RT. Oligomer-EP containing fluorescein (FAM) was prepared by dissolving FAM-labelled amylin (Bachem, Bubendorf, Switzerland) directly in NaOH/PBS, then left to aggregate, together with normal amylin in a ratio of 1 to 5, according to the oligomer protocol mentioned earlier. Before cell experiments, 100 μM fibril-EP, oligomer- EP and FAM-labelled oligomer-EP were diluted to a final working concentration of 10 μM in pericyte medium. This concentration is commonly used when investigating toxicity of amylin.^15–18^ Purity of preparations was analysed using Thioflavin T (ThT) assay and Western blotting (Supplementary Figure S2).

### HBVP treatment

The HBVPs were stimulated with cell culture medium containing 10 μM oligomer-EP and fibril-EP or FAM-labelled oligomer-EP. Treatment with NaOH/PBS was used as fibril/oligomer control. The cells were incubated in 37℃ for 3–24 h. Cell culture supernatant was collected and centrifuged (275 × *g* for 5 min at 4℃) after treatment. Cells were lysed using a cell lysis kit (Sigma-Aldrich, St. Louis, MO). Protein concentrations in wells exposed to different stimuli were verified using a bicinchoninic acid (BCA) assay kit (Sigma-Aldrich, St. Louis, MO).

### Lactate dehydrogenase/cytotoxicity assay

Cell toxic properties of amylin after 3, 6, 12, 18, and 24 h exposure to oligomer-EP, fibril-EP amylin or their control were analysed using lactate dehydrogenase (LDH) assay according to manufacturer’s instructions (Sigma-Aldrich, St. Louis, MO). The basic principle of this widely used cell toxic assay is based on the fact that LDH leaks into the medium when the plasma membrane disrupts in response to cell death. Since LDH catalyses the interconversion of with concomitant NADH and NAD^+^, it is possible to quantify LDH activity by measuring the rate of decrease in absorbance when the LDH reduces NAD to NADH. Cell culture supernatant from cells treated with 1 μM staurosporine (Sigma-Aldrich, St. Louis, MO) was used as positive control for cytotoxicity. The cell experiment was performed in triplicate at each time point.

### Immunocytofluorescence staining

The HBVPs were rinsed with ice-cold PBS and fixed with 2% formaldehyde for 15 min followed by incubation in blocking solution (5% goat serum in PBS) for 30 min at RT. The HBVPs were incubated with mouse anti-NG2 (1:400, Millipore) in blocking solution. Cells were incubated for 2 h at RT in the dark with secondary antibodies goat anti-mouse Cy3 (1:250, Jackson ImmunoResearch, West Grove, PA). Vectashield Set mounting medium with DAPI was used to mount the cells. Changes in nuclei morphology were investigated by analysing pictures (captured at 20× magnification) of untreated HBVPs, control HBVPs and HBVPs exposed to oligomer-EP or, fibril-EP amylin for 24 h. Percentage of nuclei with altered morphology was determined as number of altered (kidney shaped, blebbed or fragmented) cell nuclei divided by total number of cell nuclei (DAPI-positive cells). On average 350 cells/stimuli in three individual experiments were analysed by two observers blind to the stimulation.

### Caspase 3/7 activity assay

Caspase 3/7 activity in HBVP after 3, 6, 12, 18 and 24 h exposure to oligomer-EP, fibril-EP amylin or their control was analysed using Caspase-Glo 3/7 luminescence assay according to the manufacturer’s instructions (Promega, Fitchburg, WI). The cell experiment was performed in triplicate at each time point.

### NG2 analysis

Levels of NG2 in HBVP lysate were analysed using an in house developed assay based on Meso Scale Discovery electrochemiluminescence technology and their immunoassay conversion kits (Meso Scale Discovery, Rockville, MD). The procedure has been described previously.^13,19^ The electrochemiluminescence signal was quantified using a Meso Scale Discovery SECTOR Imager 6000. The NG2 levels were analysed in lysates obtained from three separate experiments with three replicates for each experiment. Protein concentration in cell lysates was analysed using a BCA Protein Assay Kit according to manufacturer’s instructions. Analysis of protein concentrations showed no differences between the stimulated wells (data not shown).

### Analysis of amylin internalization

Intracellular amylin distribution was analysed by stimulating HBVP with oligomer-EP containing FAM-labelled amylin for 14 h. Amylin distribution in relation to lysosomes was investigated by adding Lysotracker Red DND-99 (Invitrogen, Carlsbad, CA) (a fluorescent acidiotropic probe highly selective for acidic organelles) to the cells for the last 2 h of amylin incubation. Cellular localization of oligomer FAM-labelled amylin and co-localisation percentage (Mander’s coefficient) between oligomer FAM-labelled amylin and Lysotracker Red DND-99 or DAPI-stained cell nuclei in HBVPs (n = 10) were analysed using confocal microscopy equipped with a 63×objective and Zen 2009 software.

### Autophagy

Alterations in autophagosome formation in response to amylin were analysed using BacMam LC3B-FP (Invitrogen, Carlsbad, CA), an LC3 fluorescent protein (FP) chimera in combination with high transduction efficient BacMam (insect baculovirus with a mammalian promotor). HBVPs exposed to oligomer-EP amylin, fibril-EP amylin and control for 14 h or 24 h were transfected with BacMam LC3B-FP for the last 18 h of the experiment according to the manufacturer’s instructions. Lysosomes were marked by adding Lysotracker RED DND-99 to the medium for the last 2 h of the experiment. The cells were then washed, fixed with 2% formaldehyde and examined using an Olympic fluorescent microscope with a 40× objective. Changes in autophagosome formation were investigated by analysing pictures (captured at 20× magnification) of the stimulated HBVPs randomly selected in the blue channel (DAPI). On average, 500 cells/stimuli were captured after 14 h of stimuli (two individual experiments) or 24 h of stimuli (three individual experiments). Percentage of cells with increased autophagosome formation was determined as the number of HBVPs with more than 5 LC3B-FP positive dots divided by total number of cell nuclei (DAPI-positive cells). The images were analysed by two observers blind to the stimulation. Co-localisation between LC3B-FP and Lysotracker Red DND-99 in HBVPs stimulated for 14 h (n = 10/stimuli) or 24 h (n = 10/stimuli) was analysed using confocal microscopy equipped with a 63×objective and Zen 2009 software. Overlap percentage (Mander’s coefficient) of LC3B-FP and Lysotracker Red DND-99 was calculated using Zen 2009 software. Lysosomal size in HBVPs analysed by confocal was examined using Olympus cellSens Dimension. Lysosomes with a diameter larger than 3 µm (more than twice the average size of a regular lysosome) were considered as enlarged.

### Statistical analysis

Statistical analysis was performed using SPSS software (version 22 for Mac, SPSS Inc, Chicago, IL). Kolmogorov–Smirnov test showed that amylin cell inclusions were not normally distributed, and comparisons between NCs and patients with AD were therefore analysed using non-parametric Mann–Whitney test. Differences in NG2 immunoreactive intensity between amylin-containing pericytes and pericytes without amylin were analysed using paired *t* test. Paired *t* test was chosen in respect of the importance to compared NG2 intensity within each picture taken (each comprising of one amylin-containing pericyte and one pericyte without amylin) to avoid potential fading differences. Significant differences between oligomer-EP and fibril-EP amylin values and fibril/oligomer control (NaOH/PBS) values were analysed using one-way analysis of variance (ANOVA), followed by Bonferroni post hoc correction (comparisons for n = 3). Results are presented as means ± standard deviation and a value of *p* < 0.05 was considered statistically significant.

## Results

### Brain tissue studies

#### Amylin forms cell inclusions associated with microvessels

**Figure 1.**
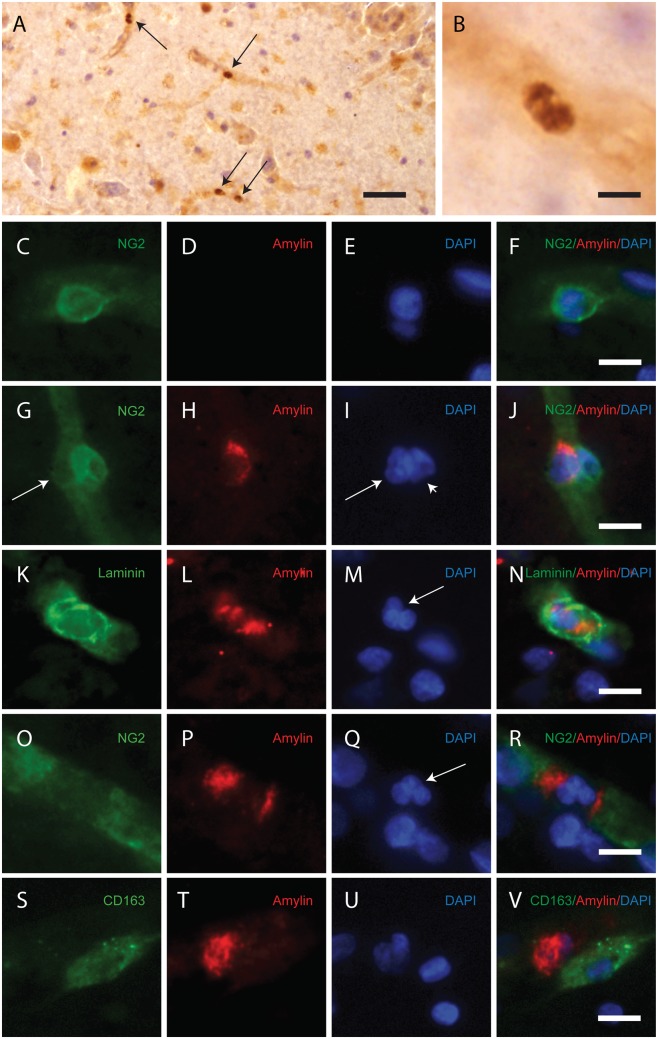
Images demonstrating immunohistological stainings of amylin and double immunofluorescence staining against NG2/amylin, laminin/amylin and CD163/amylin in the hippocampus of the patient with AD and T2D. Amylin cell inclusions are indicated with arrows in (a) and shown in a higher magnification in (b). Pericytes with round cell bodies and NG2-positive coverage of the microvessel surface (green in c), without amylin cell inclusions (red in d), displayed round DAPI-positive cell nuclei (blue in e). The images in (c), (d) and (e) are merged in (f). Cells with more diffuse and weak NG2 staining (indicated by the arrowhead, green in g) and cytosolic amylin cell inclusions (red in h) showed altered cell nuclei (indicated with arrow, blue in i). The adjacent unaffected NG2-positive cell is indicated with an arrowhead in (i). The images in (g), (h) and (i) are merged in (j). Cells enclosed by laminin (green in K) contained amylin grains (red in l) and fragmented DAPI-positive cell nuclei (indicated with an arrow blue in m). The images in (k), (l) and (m) are merged in (n). Loss of NG2 coverage (green in o) was associated with polarized amylin cell inclusion (red in p) and fragmented DAPI-positive cell nuclei (indicated with an arrow, blue in q). The images in (o), (p) and (q) are merged in (r). Staining against macrophage marker CD163 (green in s) did not co-localize with amylin cell inclusions (red in t). The cell nucleus was stained with DAPI (blue in U). The images in (s), (t) and (u) are merged in (v). Scale bars: (a) 50 µm, (b), (c) to (v) 5 µm.

To investigate amylin cell inclusions associated with microvessels in human tissue, we stained sections from hippocampus and PHC of NCs and AD patients against amylin. In line with previous studies,^1^ we found amylin cell inclusions (Figure 1(a) and (b)) associated with microvessels in all individuals. To estimate potential differences in number of amylin cell inclusions between NCs and AD patients, we counted cell inclusions in multiple fields sampled from the hippocampal ML and the III–IV cortical layer of PHC. Both PHC and ML were chosen because microvessels in these areas are easy to detect. No difference between AD patients and NCs in terms of the number of amylin cell inclusions was noted in either hippocampus (*p* = 0.537) or PHC (*p* = 1.000), but the two individuals with T2D had more than double the number of cell inclusions. Difference in amylin cell inclusions between AD and NCs remained non-significant in both hippocampus (*p* = 0.286) and PHC (*p* = 1.000) after omitting the two T2D values. We were unable to distinguish amylin aggregates/plaques in the brain parenchyma or in the vessel walls, and we found no amylin cell inclusions outside microvessels.

#### Amylin cell inclusions are associated with altered cell nucleus morphology and reduced NG2 expression

We further identified the amylin-containing cells as pericytes by immunofluorescence staining against amylin together with the pericyte marker NG2^20^ and laminin (Figure 1 and Supplementary Figure S2). The latter marker reveals the basal lamina, which encloses pericytes, but only delineates the abluminal side of endothelial cells.^21^ Pericytes not associated with amylin cell inclusions were visualized as complete coverage of microvessels by NG2-positive staining, round NG2-positive cell bodies and round DAPI-positive cell nuclei (Figure 1(c) to (f)). The amylin cell inclusions were seen as amylin grains enclosed by NG2 (Figure 1(g) to (j) and Supplementary Figure S1(a) to (d)) and laminin (Figure 1(k) to (n) and Supplementary Figure S1(e) to (h). The amylin grains were located in the cytosol of NG2-positive cells with altered cell nucleus morphology (Figure 1 and Supplementary Figure S3), varying from slightly changed/blebbed to fragmented. Pericytes with amylin cell inclusions were often found adjacent to other pericytes and displayed weaker NG2 staining compared with unaffected pericytes (Figure 1(g) to (j). To determine the weakened NG2 staining in amylin-containing pericytes, we quantified the NG2 immunoreactive intensity in a number of amylin-containing pericytes (n = 20) and pericytes without amylin (n = 20). In line with our observation, we found significantly reduced NG2 intensity in pericytes containing amylin compared to pericytes without amylin (17.84 ± 9.05 % less NG2 intensity, p < 0.0001).

We further detected a minor number of amylin cell inclusions associated with lost NG2 coverage. These cells showed scattered and polarized cytosolic amylin grains and fragmented cell nuclei (Figure 1(o) to (r)). Staining against the macrophage-specific marker CD163^22^ showed that the number of perivascular macrophage cells was low (less than 15/section in ML and 9/section in PHC) and none of them were associated with amylin cell inclusions (Figure 1(s) to (v)). Double immunofluorescence staining against amylin and Aβ showed no co-localization between the peptides either in brain parenchyma or in microvessels (Supplementary Figure S4).

### Cell culture studies

#### Pericyte viability is decreased in response to oligomer and fibril amylin

**Figure 2.**
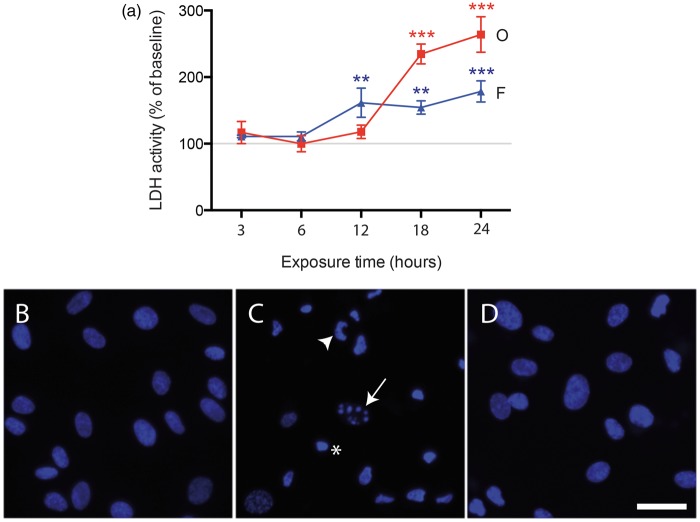
Curves demonstrating changes in LDH activity (a) in medium from HBVPs exposed to 10 μM oligomer-EP of amylin (O, red) and 10 μM fibril-EP of amylin (F, blue) compared to control (indicated by the grey line at 100%) for 3, 6, 12, 18 and 24 h. Values at each time point were analysed using ANOVA, followed by the Bonferroni post hoc correction (comparisons for n = 3). Each point represents the mean ± SD. Significant difference at *p < 0.05, **p < 0.01, ***p < 0.001. Images in (b–d) show representative images of DAPI-positive cell nucleus of control HBVPs (b), oligomer-EP (c) and fibril-EP amylin (d). Fragmented (arrow), blebbed (arrowheads) and shrinked (asterix) cell nucleus is indicated in c. Scale bar = 10 µm in (b–d).

To verify our findings and to further investigate whether the changes in pericytes observed in the brain tissue studies are a direct effect of amylin, we performed in vitro studies using primary HBVPs. Initially, we analysed cell viability by measuring LDH activity after 3, 6, 12, 18 and 24 h of exposure to oligomer- and fibril-EP amylin. We found that both oligomer- and fibril-EP significantly increased HBVP death after 18 and 12 h, respectively (Figure 2(a)). Furthermore, the response evoked by oligomer-EP was stronger compared with fibril-EP (Figure 2(a)). The LDH activity in controls did not differ significantly from LDH activity in untreated cells (*p* = 0.095) 24 h after experimental start-up. To verify the found amylin-induced cell death, we analysed cell nuclei morphology of HBVPs stimulated for 24 h. The percentage of HBVPs displaying shrinked, blebbed or fragmented nuclei was significantly increased after oligomer-EP stimulation compared to control HBVPs (73.3 ± 25.3% vs. 8.0 ± 4.3%, *p* = 0.002) (Figure 2(b) and (c)). Slightly enhanced number of HBVPs with altered cell nucleus morphology was also found after fibril-EP exposure (10.6 ± 7.2%) (Figure 2(d)), but the increase was not significant. Staining against NG2 revealed slightly altered cell morphology after oligomer-EP stimulation observed as curled up feature and/or ruffed appearance (Supplementary Figure S5).

#### Caspase 3/7 activity and NG2 lysate levels are altered in response to oligomer amylin

**Figure 3.**
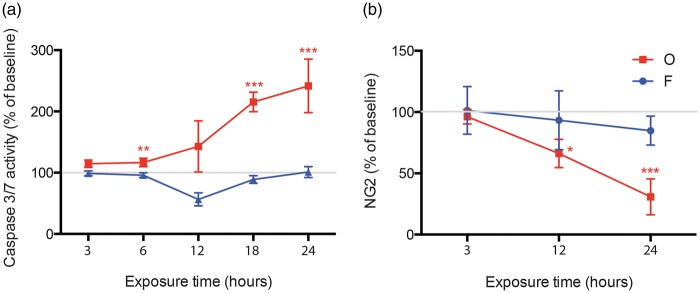
Curves demonstrating changes in caspase 3/7 activity (a) and NG2 levels (b) in lysates from HBVPs exposed to 10 μM oligomer-enriched amylin preparations (O, red) and 10 μM fibril-enriched amylin preparations (F, blue) compared to control (indicated by the grey line at 100%). Values at each time point were analysed using one-way analysis of variance (ANOVA), followed by the Bonferroni post hoc correction (comparisons for n = 3). Each point represents the mean ± SD. Significant difference at *p < 0.05, **p < 0.01, ***p < 0.001.

To investigate if amylin-induced HBVP death is caspase dependent, we analysed alterations in caspase 3/7 activity after 3, 6, 12, 18 and 24 h exposure to the two different amylin preparations. We found significantly increased caspase 3/7 activity in HBVPs exposed to oligomer-EP after 3 h, and after 24 h the activity was 2.5 times higher than the activity in control HBVPs (Figure 3(a)). In contrast, although a significant decrease in caspase 3/7 activity was noted after 12 h, the activity was overall unchanged during the 24 h exposure to fibril-EP (Figure 3(a)). Since our brain tissue study showed decreased NG2 intensity in pericytes with amylin cell inclusions, we further analysed levels of NG2 in lysates from HBVPs stimulated with the different amylin preparations for 3, 12 and 24 h. We found that while fibril-EP left NG2 lysate levels unaltered, oligomer-EP significantly decreased NG2 lysate levels at 12 h and 24 h (Figure 3(b)).

#### Internalized amylin is primarily distributed in the cytosol and co-localizes with lysosomes

**Figure 4.**
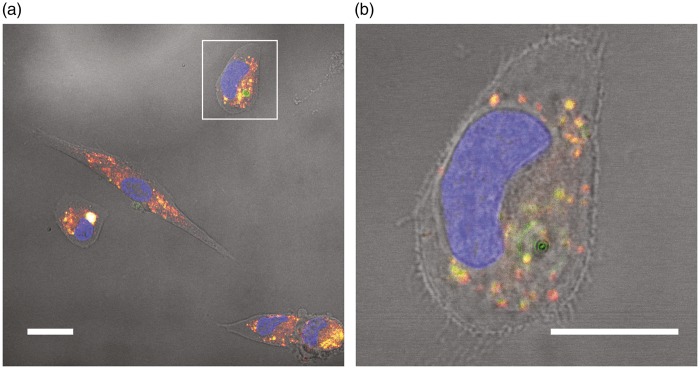
Images demonstrating amylin internalization and co-localization with lysosomes in HBVPs. Image in (a) shows HBVPs stimulated with 10 μM oligomer-enriched preparations containing FAM-labelled amylin for 14 h. Co-localization between FAM-labelled amylin (green) and Lysotracker Red DND-99-stained lysosomes (red) is visualized as yellow/white. Image in (b) shows a higher magnification of the HBVP indicated by the white square in (a). FAM-labelled amylin (green) co-localizing with lysosomes (red) is seen as yellow. Scale bars: (a) 20 µm, (b) 15 µm.

Next, we investigated pericytic amylin internalisation by analysing the intracellular distribution of amylin in HBVPs stimulated with oligomer-EP of FAM-labelled amylin. Our analysis showed that 97.7 ± 5.9% of total amylin was found outside the DAPI-stained nuclei. Moreover, 86.1 ± 10.7% of the total intracellular FAM-labelled amylin co-localised with lysosomes marked with Lysotracker Red DND-99 (Figure 4(a) and (b)).

#### Amylin affects autophagosome formation in an aggregation-dependent manner

**Figure 5.**
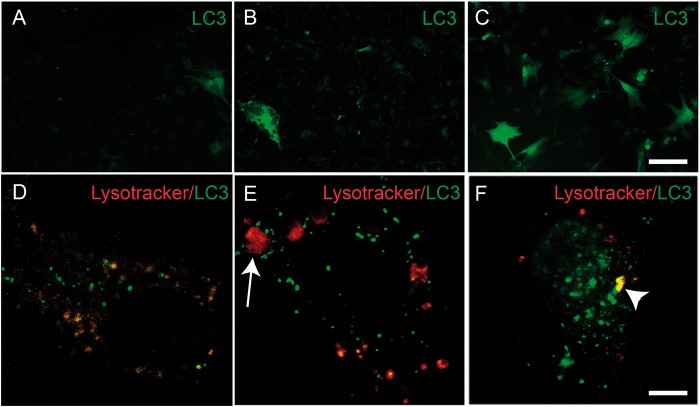
Images demonstrating autophagy in HBVPs exposed to 10 μM oligomer-enriched preparations, 10 μM fibril-enriched preparations and their control for 24 h. Images captured at 10 × magnification (a–c) demonstrate the low presence of HBVPs containing LC3B-FP marked autophagosomes (green) after treatment with control (a) and oligomer-enriched preparation (b). In contrast, fibril-enriched preparations enhanced the number of HBVPs with LC3B-FP marked autophagosomes (c). Images captured at 63 × magnification (d–e) demonstrate the localisation of LC3B-FP (green) and Lysotracker Red DND-99 (red) in HBVPs exposed to control (d), oligomer-enriched amylin preparations (e) and fibril-enriched amylin preparations (f) Co-localization between LC3-FP and Lysotracker Red DND-99-stained lysosomes is visualized as yellow/orange. Scale bar = 40 µm (a–c), Scale bar, 5 µm (d–f).

Lastly, we investigated the impact of amylin on autophagy, a cellular quality control removing abnormal, dysfunctional organelles and toxic proteins.^17^ Autophagy is initiated by the formation of autophagosomes, characterised by the incorporation of LC3 protein into the phagophore membrane. Analysis of LC3B-FP in our cultures showed that the percentage of HBVPs containing enhanced number of autophagosomes was very low after control or oligomer-EP stimulation for 14 h (1.3 ± 1.3% or 1.4 ± 0.9%, respectively) and 24 h (1.3 ± 0.5% or 0.7 ± 0.4%, respectively) (Figure 5(a) and (b)). In contrast, fibril-EP stimulation increased the percentage of HBVPs with elevated autophagosome formation already after 14 h (5.6 % ± 2.0%) and the percentage was further drastically and significantly increased after 24 h (12.9 ± 16.1%, p = 0.021) (Figure 5(c)). Since autophagy is considered to be functional when formed autophagosomes fuse with lysosomes,^23^ we also analysed the co-localisation between LC3B-FP and the lysosome marker Lysotracker Red DND-99. Confocal analysis revealed a rather low co-localisation ratio after 14 h stimulation of control (17.8 ± 11.5%), oligomer-EP (9.6 ± 7.7%) and fibril-EP (17.6 ± 13.2%) as well as after 24 h stimulation of control (2.2 ± 1.8%), oligomer-EP (1.7 ± 1.8%) and fibril-EP (4.8 ± 5.7%). Although the mean ratio was almost halved after 14 h of oligomer-EP stimulation compared to fibril-EP and control stimulation, the difference remains non-significant, and none of the other stimuli induced significant changes in co-localisation ratio regardless of time. The confocal analysis also revealed enlarged lysosomes in most of the 24 h oligomer-EP (more than 5 enlarged lysosomes in 10 out of 10 cells) and fibril-EP (more than 1 enlarged lysosome in 7 out of 10 cells) stimulated HBVPs, whereas none of the analysed control HBVPs (n = 10) had enlarged lysosomes (Figure 5(d) to (f)).

## Discussion

The aim of this study was to investigate the cellular localization of amylin cell inclusions in microvessels and determine the consequences thereof. Using immunohistological staining of hippocampus and PHC from AD patients and NCs, we show that microvessel-associated amylin cell inclusions are found in pericytes. Further analysis revealed that all amylin-containing pericytes displayed blebbed or fragmented cell nuclei, a well-known sign of cell death.^24^ The observed nuclei changes, varying from slightly changed to fragmented, were seen in all individuals, regardless of diagnosis. In view of these observations, we hypothesized that pericyte dies in response to a gradual intracellular accumulation of amylin.

A few cells with amylin cell inclusions, all with fragmented nucleus, were associated with loss of NG2 coverage. This finding is divergent as NG2-positive pericytes are usually wrapped around microvessels providing homogeneous staining of NG2 vessels. The NG2 negative cells could be pericytes with complete NG2 downregulation possibly induced by amylin. They could also be cells with another identity, e.g. endothelial cells. However, all confocal analysed amylin cell inclusions were surrounded by laminin (basal lamina), again indicating a pericyte identity. Still, since not all amylin cell inclusions in all individuals were analysed, we are unable to fully exclude the possibility that amylin also can form cell inclusions in endothelial cells. We also investigated whether amylin cell inclusions can be found within perivascular macrophages, immune-cells associated with microvessels. Staining against the perivascular macrophage marker CD163^22^ showed, however, that none of the cells with amylin cell inclusions were CD163 positive.

As the altered cell nuclei found in amylin-containing pericytes could imply a toxic property of amylin, we conducted an in vitro study where we stimulated primary HBVPs with preparations of oligomer or fibril amylin. Our LDH activity analysis as well as the analysis of cell nucleus morphology showed that both oligomer- and fibril-EP exposure induce pericyte cell death, but that oligomer-EP stimulation induce a much stronger response. This finding corresponds well with previous studies on other cell types, including neurons and β cells, suggesting that amylin, in particular the oligomeric form, is a potent toxic peptide.^25,26^ The fast and strong response to oligomer and fibril amylin is interesting considering an earlier study on pericyte viability in which we showed that 24 h exposure to 10 μM oligomer-EP Aβ42 preparations (i.e. the same concentrations as used in the present study) had no impact on pericyte viability.^19^ Hence, amylin appears to be a more toxic to pericytes than the kindred amyloid peptide commonly associated with AD pathology.

The analysis of amylin cell inclusions showed that amylin grains were found primarily in the cytosol of pericytes. Similar cellular distribution of amylin was seen in HBVPs stimulated with oligomer-EP, where most of the cytosolic amylin co-localized with lysosomes. Cellular distribution of amylin taken up by pericytes has not been reported previously, but studies have shown a similar distribution pattern of amyloid β sheet and lysosomal activity in vascular mural cells stimulated with Aβ.^27^ The co-localization between FAM-labelled amylin and lysosomes suggests that internalized amylin is taken care of by a cellular quality control mechanism. Since autophagy is known to be vital for degrading toxic molecules and damaged organelles,^17^ we found it interesting to examine how the different amylin aggregation forms affect pericyte autophagy. Autophagy function is commonly investigated by the use of fluorescently labelled LC3 (LC3-FP), an autophagosome marker. Low number of LC3-FP marked autophagosomes indicates a well-functioning autophagy as fusion with lysosomes also degrades the fluorescence protein conjugated with LC3.^28^ High number of LC3 marked autophagosomes instead indicates an increased demand of autophagy and/or an insufficient fusion with lysosomes. In regard of this, it is likely that the increased number of autophagosome-containing pericytes seen after fibril-EP amylin stimulation reflects an accumulation of intracellular toxic waste (toxic amylin and/or damaged organelles). Notably, the LC3/Lysotracker co-localisation ratio in fibril-EP-treated cells did not differ compared to control cells. It could thus be hypothesised that the increased number of autophagosomes does not reflect an altered fusion rate, but rather that the lysosomal activity does not keep up with the autophagosome formation. Interestingly, we found very few autophagosome-containing pericytes after both 14 and 24 h of oligomer-EP amylin stimulation. Moreover, although non-significant, the co-localisation ratio between LC3 and Lysotracker in oligomer-EP-treated pericytes was after 14 h almost halved compared to control and fibril-EP stimulated pericytes. It may thus be hypothesised that oligomer-EP amylin interrupts both formation of the protective autophagosomes (thereby exposing the cytosol to toxic waste) and the fusion with lysosomes. In addition, we noted several enlarged lysosomes in oligomer-EP and fibril-EP-treated pericytes. Since enlarged lysosomes indicate irreversible cell injury,^29^ we interpret these findings as yet another sign for dysfunctional lysosomal activity. In all, our results indicate that aggregated amylin affects autophagy in pericytes. This finding is in line with previous studies demonstrating disrupted autophagy in INS-1 cells,^30^ and pancreatic rat β cells^4^ after exposure to aggregated and oligomer amylin, respectively. Moreover, since inhibition of autophagy has previously shown to enhance cell death after exposure to aggregated amylin,^31^ we speculate that the impaired autophagy seen in our culture studies may in part underlie the amylin-induced pericyte death.

The NG2 staining intensity of pericytes with amylin cell inclusions was weaker compared with pericytes without amylin cell inclusions, and analysis of lysates from oligomer-stimulated pericytes showed significantly decreased NG2 levels. Hence, both our brain tissue study and in vitro study indicate that amylin affects NG2 expression. Importantly, as our BCA analysis showed no difference in the total amount of protein between the different wells after stimuli, we exclude the possibility that the reduced NG2 levels are due to reduced protein levels caused by cell death. Still we cannot exclude the possibility that the reduced NG2 detection in our assay could be due to oligomer amylin-induced fragmentation/alteration of the NG2 proteoglycan, which may interfere with or remove the antibody-binding site of NG2. Nevertheless, although the significance of oligomer amylin-induced NG2 loss is yet to be defined, it is interesting to note that the decrease in NG2 levels in our cell cultures preceded the increase in LDH activity. It may thus be that downregulation of NG2 expression is involved in oligomer-induced pericyte death. A recent study has shown that NG2 protects oligodendrocyte progenitors (OPCs) (the only other NG2 expressing brain cell type) against oxidative stress by interacting with mitochondrial serine protease OMI/HtrA2.^32^ This serine protease is translocated from the mitochondria to the cytosol in response to oxidative stress. There it degrades inhibitors of apoptosis proteins (IAPs), which in turn result in caspase 3 activation and induction on apoptosis. The oligodendrocyte study showed that the cytosolic tail of NG2 binds to OMH/HtrA2 and hinders it from degrading IAPs. The study further showed that caspase 3 activity was increased in OPCs isolated from NG2 knockout mice and pyknotic cells were evident after oxidative stress, a finding not detected to the same extent in cells isolated from wild-type mice. A similar protective role of NG2 against oxidative stress has been shown in a study reporting that human glioma cells expressing high levels of NG2 were less sensitive to oxidative stress than cells with low levels of NG2.^32^ Given these findings, it is interesting that our in vitro studies showed increased caspase 3/7 activity after 12 h exposure to oligomer-EP amylin, when NG2 lysate levels were also decreased. In contrast, no changes in caspase 3/7 activity were found after exposure to fibril-EP amylin, a condition also yielding unchanged levels of lysate NG2. In view of these findings, it is tempting to speculate that oligomer-EP, by reducing the presence of NG2, makes the cell more vulnerable to responses evoked by oxidative stress. More extensive experimental studies are however required to pinpoint the exact role for NG2 in amylin-induced pericyte death.

The significance of our findings should be viewed from the perspective that pericytes play an important role in the maintenance of microvessel integrity, BBB function, angiogenesis and removal of neurotoxic substances (including Aβ).^33^ Loss of pericyte coverage induced by accumulating intracellular amylin oligomers would thus theoretically lead to microvascular changes, which could have a tremendous impact on the brain. This idea has gained attention within the AD research field since many AD patients display enhanced BBB permeability, leakage of microvessels and altered angiogenesis, events related to pericyte function.^34,35^ It has therefore been hypothesized that pericyte loss may be involved in the pathogenesis of AD.^36^ Indeed, neuropathological analysis demonstrates a 60% reduction in brain pericytes in AD patients and this loss of pericytes was associated with increased BBB permeability and Aβ plaque load.^37^ Whether amylin oligomers are involved in AD-related pericyte loss remains to be investigated and our studies showed no difference in number of amylin inclusions between AD patients and NCs. However, it should be stressed that brain IHC staining only visualizes a snapshot of brain processes at the time of death. We are therefore unable to tell whether the AD patients had a high number of amylin cell inclusions at earlier stages of the disease or whether the low number of amylin cell inclusions reflects loss of pericytes. Another limitation of our study is the small cohort size; this study should be replicated in a larger cohort.

Lastly, one NC and one AD patient showed double the number of amylin cell inclusions compared with the other individuals. This increase could potentially be explained by the fact that both individuals had T2D, as amylin accumulation and deposition are commonly found in peripheral organs of T2D patients. It is thus tempting to speculate that increased number of cell inclusions reflects increased circulating peripheral aggregated amylin related to T2D pathology. Because amylin cell inclusions are associated with pericyte death, our findings further suggest that pericyte loss could be a prominent event in the brain of T2D patients, which may underlie the increased risk for dementia in these patients.^38,39^ This observation has to be confirmed in a larger T2D patient cohort before any justified conclusions can be drawn.

Finally, previous studies have demonstrated the presence of amylin in Aβ-positive vessels (i.e. CAA) as well as Aβ plaques in brain parenchyma.^1^ The co-localization suggests that amylin and Aβ interact, a hypothesis supported by in vitro studies demonstrating cross seeding between the two peptides.^40^ The individuals included in the current study lacked CAA pathology, and we are thus unable to confirm the previous amylin/CAA findings. Moreover, although most individuals showed Aβ plaques in both hippocampus and PHC, we were unable to distinguish amylin deposition in the brain parenchyma of AD patients or NCs. However, we used a brain tissue fixation protocol optimized for NG2 staining and thus our pretreatment of brain samples differed from the pretreatment used in the previous study. Thus, we cannot exclude the possibility that amylin deposition in the brain parenchyma may be lost in brain tissue fixed according to our protocol. We further found no co-localization between microvessel-associated amylin cell inclusions and Aβ. The lack of co-localization speaks against the idea that amylin cell inclusions in pericytes are formed by cross seeding between amylin and Aβ.

In conclusion, by using both brain tissue and in vitro studies, we show that aggregated amylin has a strong impact on the survival of brain pericytes. We also show that that amylin affects cultured pericytes in an aggregation-dependent manner. Oligomer-induced cell death is associated with increased caspase activity 3/7, loss of surface-bound protective NG2 and impaired autophagosome formation, whereas fibril-induced cell death appears to be independent of caspase 3/7 activity and NG2 loss, but associated with increased autophagosome formation. Our findings should be viewed from the fact that pericyte loss has been implicated in AD and T2D pathogenesis and thus encourage future studies investigating the potential role for amylin in dementia progression.

## Supplementary Material

Supplementary material

Supplementary material

Supplementary material

Supplementary material

Supplementary material
