# Supplementary figures and images for: Amylin alters human brain pericyte viability and NG2 expression
Source: J Cereb Blood Flow Metab. 2016 Jan 1;37(4):1470–82. doi: 10.1177/0271678X16657093 (PMC5453466; doi:10.1177/0271678X16657093)

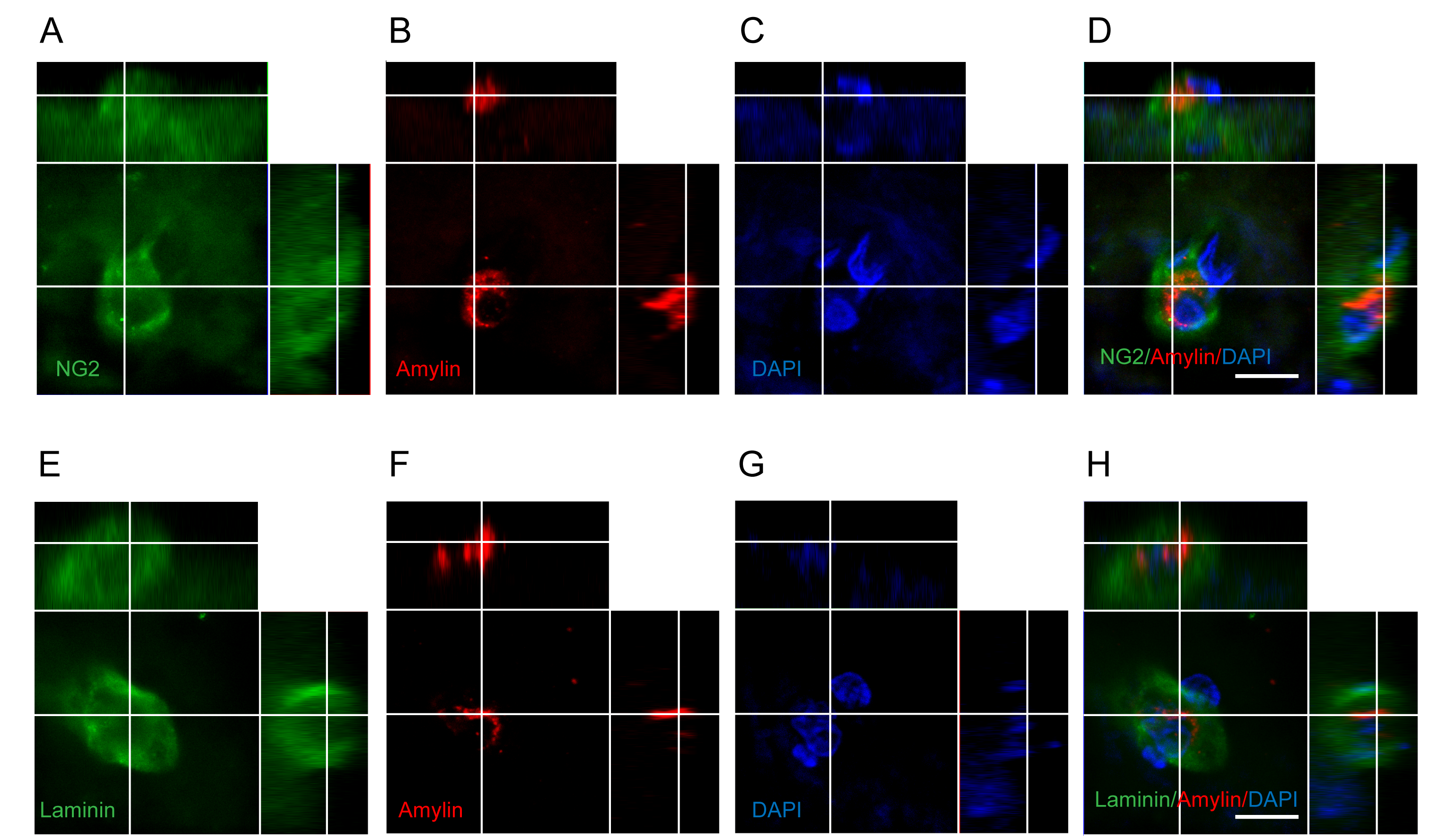

Supplement: Supplementary material [file Figure1_093.tif]

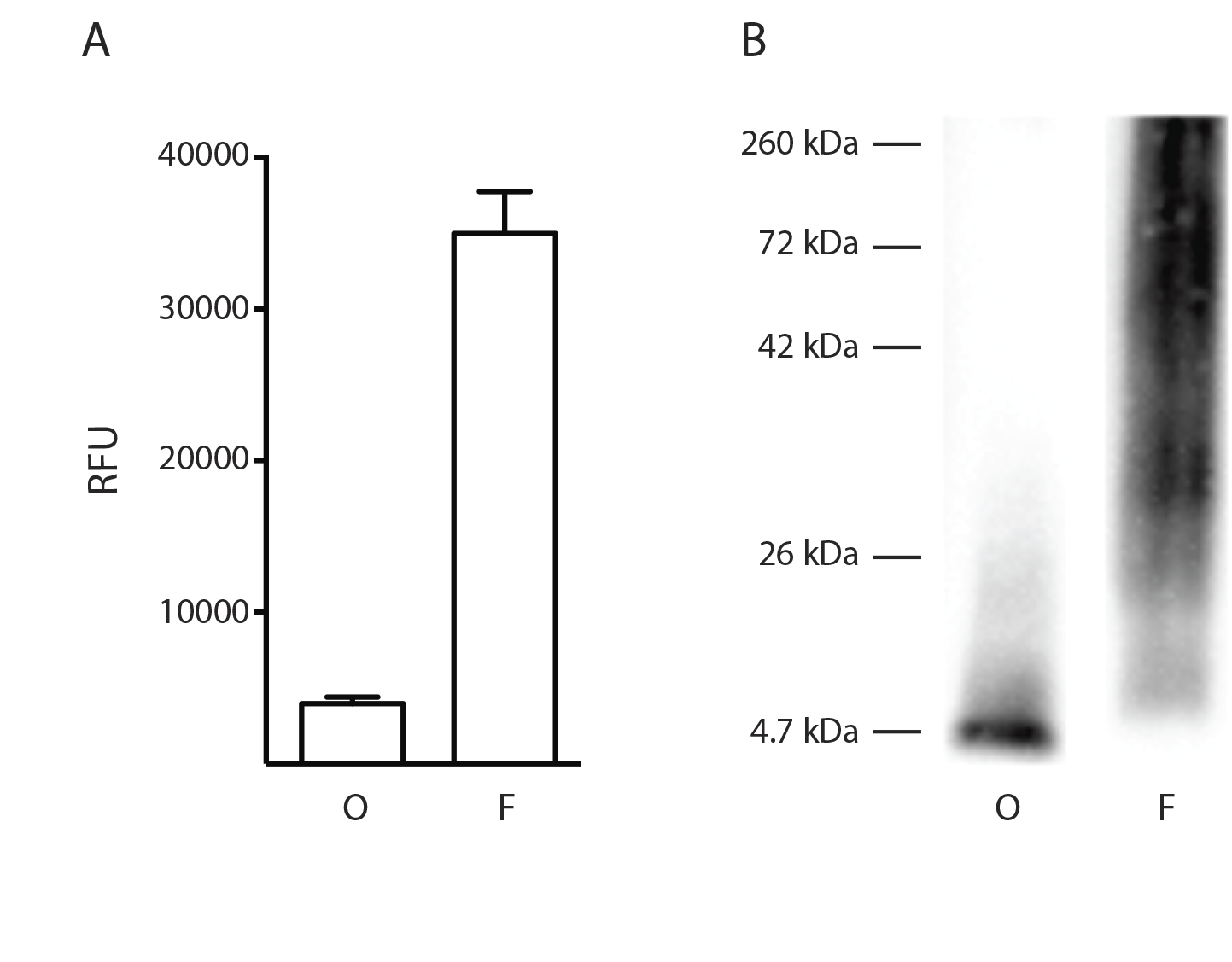

Supplement: Supplementary material [file Figure2_093.tiff]

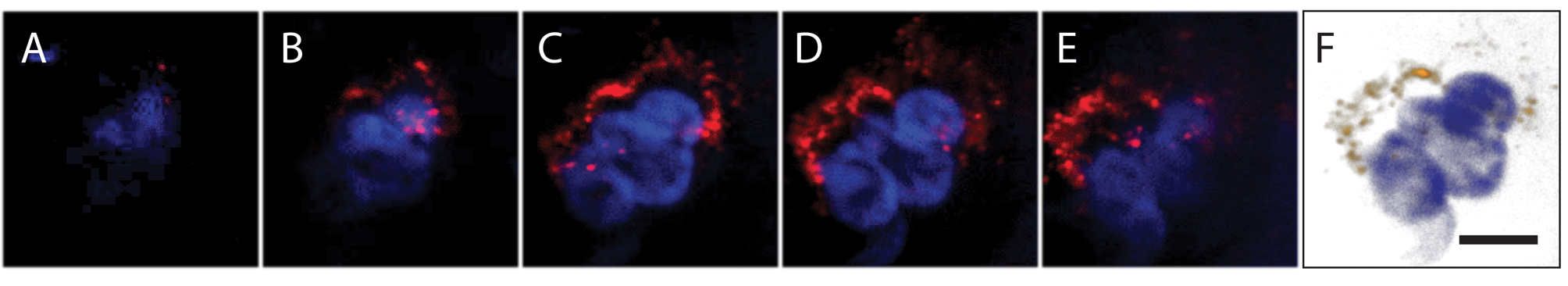

Supplement: Supplementary material [file Figure3_093.tif]

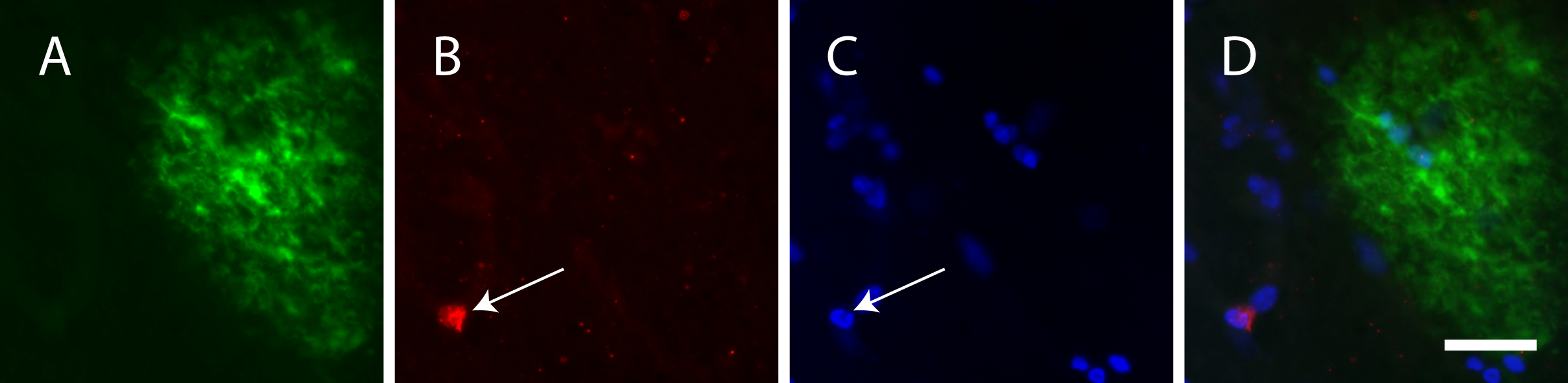

Supplement: Supplementary material [file Figure4_093.tif]

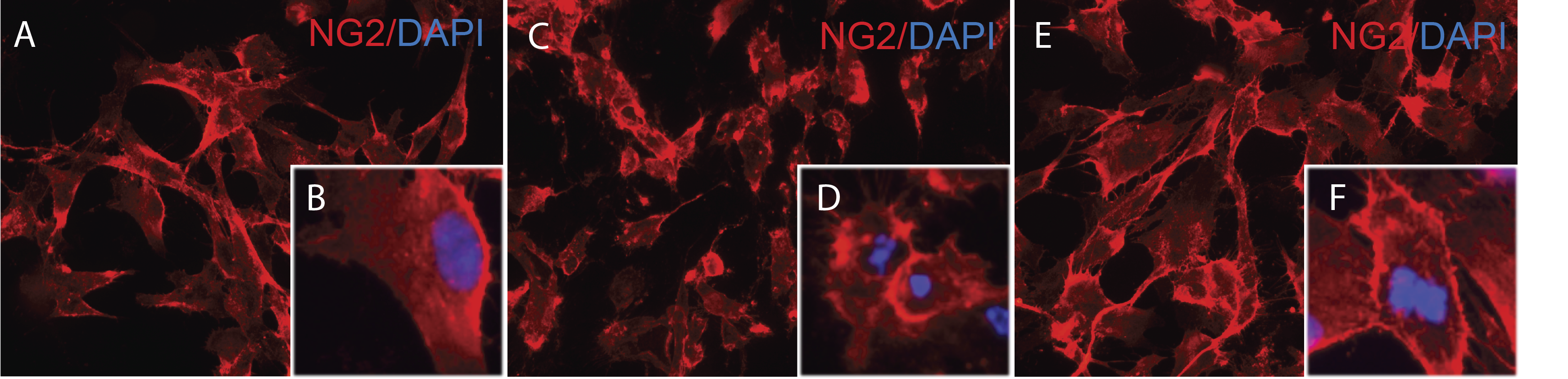

Supplement: Supplementary material [file Figure5_093.tiff]
